# A retrospective report (2003–2013) of the complications associated with the use of a one-man (head and tail) rope recovery system in horses following general anaesthesia

**DOI:** 10.1186/s13620-018-0117-1

**Published:** 2018-02-13

**Authors:** Maria Chie Niimura del Barrio, Florent David, J. M. Lynne Hughes, David Clifford, Hans Wilderjans, Rachel Bennett

**Affiliations:** 1 UCD Veterinary Hospital, University College Dublin, Belfield, Dublin 4 Ireland; 2 Equine Veterinary Medical Center, Al Shaqab- a member of Qatar Foundation, PO Box 90005, Al Shaqab Street, Al Rayyan, Doha, Qatar; 3 Dierenkliniek De Bosdreef, Spelonckvaart 46, 9180 Moerbeke-Waas, Belgium; 4 The Granary, Bunstead Barns, Poles Ln, Hursley, Winchester, SO21 2LL UK

**Keywords:** Anaesthesia, Rope-assisted recovery, Complications, Equine, Mortality

## Abstract

**Background:**

The mortality rate of horses undergoing general anaesthesia is high when compared to humans or small animal patients. One of the most critical periods during equine anaesthesia is recovery, as the horse attempts to regain a standing position. This study was performed in a private equine practice in Belgium that uses a purpose-designed one-man (head and tail) rope recovery system to assist the horse during the standing process.

The main purpose of the retrospective study was to report and analyse complications and the mortality rate in horses during recovery from anaesthesia using the described recovery system. Information retrieved from the medical records included patient signalment, anaesthetic protocol, duration of anaesthesia, ASA grade, type of surgery, recovery time and complications during recovery. Sedation was administered to all horses prior to recovery with the rope system. Complications were divided into major complications in which the horse was euthanized and minor complications where the horse survived. Major complications were further subdivided into those where the rope system did not contribute to the recovery complication (Group 1) and those where it was not possible to determine if the rope system was of any benefit (Group 2).

**Results:**

Five thousand eight hundred fifty two horses recovered from general anaesthesia with rope assistance. Complications were identified in 30 (0.51%). Major complications occurred in 12 horses (0.20%) of which three (0.05%) were assigned to Group 1 and nine (0.15%) to Group 2. Three horses in Group 2 suffered musculoskeletal injuries (0.05%). Eighteen horses (0.31%) suffered minor complications, of which five (0.08%) were categorised as failures of the recovery system.

**Conclusions:**

This study reports the major and minor complication and mortality rate during recovery from anaesthesia using a specific type of rope recovery system. Mortality associated with the rope recovery system was low. During recovery from anaesthesia this rope system may reduce the risk of lethal complications, particularly major orthopaedic injuries.

## Background

It is well documented that equine general anaesthesia is associated with a relatively high morbidity and mortality rate. The largest equine multi-centre study [1, 2] reported an overall perioperative mortality rate at 7 days of 1.9%, as compared with that reported in small animals (0.17%) [3], and human anaesthesia (0.01%) [4]. Even in totally healthy horses, the mortality rate was 0.9%, and in horses undergoing emergency procedures it was 13.9% (5846/41824) [1, 2]. Other smaller studies report mortality rates as high as 35% and as low as 0.2% [5, 6].

It has previously been highlighted that one of the most critical periods of any anaesthetic is the recovery phase and complications arising intra-operatively, if not managed appropriately, may have a negative impact on the quality of the recovery [7]. Musculoskeletal complications such as fractures, accounted for 25.6% (84/328) and myopathies for 7% (23/328) of all anaesthetic complications in the study by Johnston et al. [2]. Recently, Dugdale et al. (2016) reported a fatal complication rate in recovery of 1% (14/1416), with fractures and dislocations accounting for 71.4% of these deaths [8]. The Dugdale study was the first to analyse factors affecting the quality of recovery. They reported that recovery quality was associated negatively with greater body mass, higher ASA (American Society of Anesthesiologists) grade, longer duration of general anaesthesia and out-of-hours procedures. All horses except for two that underwent general anaesthesia for fracture repair were recovered without assistance. These studies highlight the need to reduce the risk of recovery-associated mortality in horses.

A variety of pharmacological and physical approaches and techniques have been employed in the hope of improving recovery quality and thereby reducing the number of fatalities occurring during the recovery period. Pharmacological techniques aim to prolong recovery and allow the horse to eliminate the inhalant anaesthetic prior to regaining proprioception, and thus ensure a smoother and calmer recovery. Drugs such as alpha_2_ agonists injections, propofol or ketamine infusions in combination with alpha_2_ agonists infusion have been used; these studies have reported variable effects on recovery quality [9–12].

Santos et al. in 2003 [9] showed that the administration of low doses of the alpha_2_ agonists xylazine, detomidine or romifidine IV at the end of anaesthesia prolonged recovery time but improved the quality with less ataxia compared to a control group. The authors stated that the degree of sedation with romifidine was greater, however they did not report that the quality of recovery was better (or worse) compared to the other alpha_2_ agonists. Woodhouse et al. (2013) compared romifidine and xylazine, at two different doses, as sedatives during recovery [10]. They reported better recovery quality when the higher dose (0.02 mg/kg) of intravenous romifidine was administered. Moreover, Dugdale et al. (2016) also concluded that sedation during recovery was associated with better recovery score; a variety of alpha_2_ agonist drugs were used in this study [8]. Steffey et al. [11] investigated the effects of using xylazine (0.03 mg/kg/min) together with a bolus of propofol (0.75 mg/kg) followed by a propofol CRI (0.125 mg/kg/min) for 15 min as sedation for recovery. This combination was shown to improve the quality of transition from lateral recumbency to standing but there was also a potential increase in respiratory depression (hypoventilation, hypoxaemia and apnoea). Wagner et al. [12] failed to show any improvement of recovery quality when xylazine and ketamine CRIs, 20 μg/kg/min and 60 μg/kg/min respectively, were used for 30 min after stopping isoflurane.

The use of different forms of physical support during recovery aim to stabilize or restrain the horse until it achieves a standing position in a stable and coordinated manner. In this way, it is hoped that the risk of life-threatening musculoskeletal complications is minimised. Physical techniques include relatively simple forms of assistance such as: personnel within the recovery stall manually assisting the horse, use of a deflating air pillow mattress or pad [13]; the application of head and tail ropes [14]; and more complex systems which lift the horse or have a ‘weight neutralizing function’ such as sling recovery (Anderson Sling) [15], use of a tilt table for recovery [16], and the pool recovery systems (Hydro-pool or pool-raft system) [17, 18]. All of the above techniques offer documented advantages and potential disadvantages, but no recovery system has completely eliminated the risk of injury to the patient or the personnel who assist in recovery.

A rope-assisted recovery system was designed by clinicians at the Dierenkliniek De Bosdreef, Belgium in 1998 and has been in use since then. This recovery system was designed to improve the quality and consistency of equine recovery and thereby reduce the incidence of complications following anaesthesia and surgery. For the purpose of this study we refer to it as the one-man (head and tail) rope recovery system. This is a simple system that offers some advantages over other assisted systems because it consists of inexpensive components and requires only one person for operation since the tail rope is fixed under tension by using a mountaineering belay device. [14].

Thus far, no publication has reported morbidity and mortality rates associated with the use of a rope-assisted recovery technique in horses. The aim of our study was to report major and minor complications associated with the use of the one-man (head and tail) rope recovery system in 5852 horses following general anaesthesia. In particular we wished to isolate fatal musculoskeletal injuries, and suggest some recommendations and limitations for use of the technique.

## Material and methods

### Inclusion criteria

Anaesthetic records of horses recovered with the one-man (head and tail) rope recovery system from January 2003 until August of 2013 were reviewed and those with a complication recorded in the recovery were identified. All of the data were collected from the practice database where the recording was performed contemporaneously using the 4D computer software programme (Bosdreef in 4 D).

### Data retrieved

Data collected from the medical records were: patient signalment (breed, sex, age and body weight); pre-anaesthetic evaluation (i.e. heart rate, respiratory rate, capillary refill time, body temperature, packed cell volume and ASA grade of physical status (Table 1); procedural information (date of admission to the hospital, date of surgery, surgical procedure, duration of anaesthesia and duration of recovery), nature of the procedure (elective, emergency); anaesthetic protocol (premedication, induction, maintenance, analgesic techniques intra-operatively, treatment of hypotension when mean arterial blood pressure was below 70 mmHg) and description of recovery. Information regarding complications during recovery and whether or not the horse survived were retrieved from the medical files.

**Table 1:** ASA (American Society of Anesthesiologists) grade of physical status

Category	Physical Status
ASA 1	Normal healthy patient
ASA 2	Patient with mild systemic disease
ASA 3	Patient with severe systemic disease that is not a constant threat to life
ASA 4	Patient with severe systemic disease that is a constant threat to life
ASA 5	Moribund patient not expected to survive with or without surgery

### Anaesthesia monitoring

Heart rate, respiratory rate and invasive blood pressure were monitored. Arterial blood gas analyses were recorded every 30 min while electrocardiography and pulse oximetry were monitored continuously and recorded every ten minutes throughout anaesthesia. If mean arterial blood pressure dropped below 70 mmHg, dobutamine infusion was administered intravenously (IV) to effect, commencing at 0.5 μg/kg/min. Horses were mechanically ventilated using a large animal ventilator (Mallard Large Animal Anaesthetic Model 2800 C, Mallard Medical, Redding CA) and tidal volume, peak inspiratory pressure and respiratory rate were adjusted to maintain normocapnia (end tidal carbon dioxide between 35 and 45 mmHg or 4.5 and 6 kPa).

### Recovery procedure and operation of the rope recovery system:

All horses were routinely sedated in recovery with IV xylazine, butorphanol or romifidine. Selection of the sedative(s) was based on the anaesthetist’s preference. Lights were turned on or off at the discretion of the anaesthetist. The bladder of each horse was catheterised during anaesthesia to enable it to be emptied prior to recovery. At the end of surgery all horses were transferred to a 4.5 × 2.6 m recovery box where they were recovered with the rope recovery system. Using the hoist, the horse was positioned in lateral recumbency (parallel to the long axis of the recovery box, opposite to the doors) with the operated leg (when applicable) uppermost, and the hind quarters positioned in a corner. This positioning ensured the shortest distance from the tail knot to the tail pulley system. Using an inelastic marine rope, a self-tightening knot was then placed on the tail, just distal to the last caudal vertebra. The other end of the tail rope was run through a carabiner attached to a built-in metallic ring secured to the wall above the tail. The tail rope was then run through a mountaineering belay device positioned outside the recovery box (Grigri®, Petzl, France). The Grigri® was closed according to the manufacturer’s recommendations (where horse = climber) and secured to the outside wall via another carabiner attached to a built-in metallic ring. Finally, maximum tension was applied on the tail rope via the Grigri® in preparation for recovery. The head rope was secured to the head collar using a quick release knot, and then run through a carabiner attached to a built-in metallic ring secured to the wall above the head. The head rope was brought outside the recovery box stall to the operator’s hands. Initially, the operator maintained tension on the head collar to reduce swinging of the head and neck and prevent the horse achieving sternal recumbency. Once the operator considered that the horse had adequate strength and coordinated movements, the head rope was slightly loosened to allow the horse to use its head and neck to swing into sternal recumbency and eventually a standing position. It was important that the operator allowed movement of the head but at the same time provided some tension on the rope. Although there was no need to adjust the Grigri® during recovery, easy access to it was ensured for safety reasons. Once the horse was standing and steady, the Grigri® and then the head rope were progressively loosened until the horse could stand free. It was routine practice to maintain the recovery area as quiet as possible in order to avoid disturbing the horse during this period.

Complications recorded during recovery and any specific problems which occurred during recovery were described. Complications were subsequently divided into 2 groups: major complications where the horse died or was euthanized; or minor complications such as head/tail rope failure, facial nerve neuropathy, minor cuts, lacerations or abrasions from which the horse survived. Major complications were further subdivided into those where the rope system did not contribute to the recovery complication (Group 1) and those where it was not possible to determine if the rope system was of any benefit (Group 2).

### Descriptive statistics

Data were assessed for normality (Shapiro Wilk test) and expressed as either mean ± standard deviation (SD) or median (minimum and maximum range) where appropriate.

## Results

Over the nine and half year period, 5852 general anaesthetics were performed in which the one-man (head and tail) rope system was used to assist recovery. A total of 30 horses were identified as experiencing complications during recovery (0.51%). Details of the patient signalment, surgical procedure, anaesthetic protocol and recovery outcome are detailed below.

### Signalment

Among horses suffering complications, the breeds represented were Warmblood (24), Friesian (2) and one of each of crossbred, Belgium Riding Pony, Thoroughbred, and one horse in which the breed was not recorded. There were 17 mares, 11 geldings and 2 stallions with a mean age of 8.9 ± 4.65 years. There was one horse with no age recorded. Mean weight was 522 ± 90 kg.

### Procedure

Table 2 provides details of the surgical procedures performed in all the horses that suffered major complications during recovery. Fourteen surgeries were elective, twelve were emergencies and in four horses this information was not recorded. The median duration of anaesthesia was 98 min (range 40–300 min). The duration of one procedure was not recorded. ASA grade varied from I to IV, five horses were not assigned a status. Twelve horses were ASA I, seven were ASA II, four were ASA III and two were ASA IV.

**Table 2:** Details of major complications in horses following recovery with one-man (head and tail) rope recovery system

No	Age (years)	Gender	Procedure	Anaesthesia (minutes)	Complic	Death	Stood	Group
1	6	Mare	Ex lap	76	CPA	Sudden	No	1
2	15	Mare	Ex lap	123	CPA	Sudden	No	1
3	5	Gelding	Ex lap	194	CPA	Sudden	Yes	1
4	19	Mare	Fracture repair	300	CPA	Sudden	Yes	2
5	12	Mare	Neurectomy	76	Myopathy	Euth	Yes	2
6	9	Mare	Tooth extraction	131	Myopathy	Euth	Yes	2
7	0.8	Filly	Maxillary cyst	131	Syst dist	Euth	Yes	2
8	7	Gelding	Cast change	40	Syst dist	Euth	Yes	2
9	10	Mare	Ex lap	115	Myopathy	Euth	No	2
10	15	Mare	Ex lap	194	Joint dislocation	Euth	Yes	2
11	16	Mare	Ex lap	105	Fracture	Euth	Yes	2
12	18	Mare	Wound repair	96	Fracture	Euth	No	2

*Complic* complications, *CPA* Cardio Pulmonary Arrest, *Ex lap* Exploratory laparotomy, *Euth* euthanised, *Syst dist* Systemic disturbance

Details of the 12 horses that suffered major complications leading to death/euthanasia following general anaesthesia and recovery with the one-man (head and tail) rope recovery system. Group 1 – the rope recovery system did not contribute to the recovery complication. Group 2 – it was not possible to determine if the rope system was a contributing factor to the complication

### Anaesthetic protocol

Premedication and induction agents were recorded for 29 of the 30 horses that experienced complications. Premedication consisted of: acepromazine, butorphanol and detomidine (11 horses); detomidine (6 horses); acepromazine and romifidine (3 horses); acepromazine and detomidine (3 horses), romifidine (2 horses); and one each of xylazine and butorphanol, detomidine and butorphanol, medetomidine alone and xylazine alone. Anaesthesia was induced with ketamine and midazolam in 24 horses or with ketamine and diazepam in 5 horses. Anaesthesia was maintained in 28 horses with isoflurane in oxygen in combination with a constant rate infusion (CRI) of detomidine (15 horses), lidocaine (9 horses), medetomidine (2 horses), medetomidine combined with lidocaine (1 horse) and romifidine, ketamine and guaifenesin (1 horse). In two horses there was no record of drugs used for anaesthesia maintenance.

Analgesic drugs administered were at the anaesthetist’s discretion and comprised: morphine (7 horses), methadone (7 horses), butorphanol (1 horse) or buprenorphine (1 horse). All horses received a non-steroidal anti-inflammatory drug prior to surgery (phenylbutazone in 4 horses, flunixin meglumine in 2 horses and drug not specified in the remainder) or local analgesic block with lidocaine (1 horse) or bupivacaine (2 horses).

In 25 horses the drugs used in recovery were recorded: 21 were sedated with xylazine, two with butorphanol and one each with romifidine and butorphanol. Duration of recovery was recorded in 23 horses; median duration was 54 min (18–203).

### Recovery outcome

From the total of 5852 horses undergoing anaesthesia and recovered with rope recovery system, 30 (0.51%) suffered complications. Major complications occurred in 12 horses (0.20%) of which three were assigned to Group 1 (0.05%) and nine to Group 2 (0.15%), (Table 2), whilst minor complications occurred in 18 horses (0.31%) (Table 3).

**Table 3:** Details of minor complications in horses following recovery with the one-man (head and tail) rope recovery system following various surgical procedures

N	Complications	Additional comments	Surgical procedure
5	Equipment failure	Loose halter	Exploratory laparotomy
		Tail hair broke	Sarcoid removal
		Tail hair broke	Tooth extraction
		Tail rope slipped off	Castration
		Facial paralysis	Wound closure
4	Poor quality	Fell down after standing	Arthroscopy
		Several attempts to stand	Arthroscopy
		Lost shoe and broke hoof wall	Sarcoid removal
		None	Arthroscopy
3	Long recovery	None	Tumour removal
		None	Exploratory laparotomy
		Cardiac arrest. Resuscitated	Arthroscopy
2	Excitation	Respiratory problem	Neurectomy
		Wound to coronary band.	Desmotomy/Neurectomy
2	Myopathy	None	Exploratory laparotomy
		None	Metatarsal fracture repair
1	Restless	Dog–sitting position	Eye surgery
1	Hindlimb Weakness	None	Mandibular fracture

*N* (number of horses)

Details of the 18 horses that suffered minor (non-fatal) complications following general anaesthesia and recovery with one-man (head and tail) rope recovery system, and surgical procedures carried out

Twelve horses suffered major complications from which they died or euthanasia was necessitated (Table 2): three suffered cardiac arrest, three developed myopathies, two sustained fractures, two horses developed systemic complications (one each of metabolic acidosis and presumed ‘malignant hyperthermia’ with myopathy), one experienced respiratory arrest and one suffered an open joint dislocation. Of these 12 horses, six were anaesthetised for exploratory laparotomy due to symptoms of colic and five were mares between 15 and 19 years of age.

Three of the 12 horses (1, 2, and 3) were assigned to Group 1. Two of these horses died prior to making any attempt to stand, therefore the rope-assisted recovery system played no role in their recovery quality. Horse 1 was a six year old mare, whilst, Horse 2 was a pregnant broodmare in her tenth month of gestation. Horse 3 had a good quality of recovery; however respiratory problems during recovery were exhibited and the horse died after it stood up. No post-mortem examination was performed but laryngeal collapse was suspected.

The remaining nine horses, (equivalent to 0.15% of all recoveries during the study period) recovered from general anaesthesia but were euthanised several hours or days later due to a poor prognosis. These horses were assigned to Group 2 since the involvement of the recovery system upon the development of the condition (and subsequent euthanasia) could not readily be excluded. Only three of the horses assigned to Group 2 suffered musculoskeletal injuries (0.05%). Horse 4 (no ASA assignment) was a 19 year old mare presenting for repair of a severely comminuted articular fracture of the first phalanx and distal limb cast placement. Despite several attempts to stand with the aid of the rope recovery system, these were unsuccessful. The decision was taken to release this mare from the ropes and leave her to recover freely. The mare was assigned to Group 2 since the rope recovery system may have adversely affected recovery quality. This horse died within 24 h of surgery and is believed to have suffered from severe myopathy and cardiac arrest. The exact cause is unknown as no post-mortem was performed. Horse 5 underwent fasciotomy and neurectomy of the deep branch of the lateral metatarsal nerve. The mare developed myopathy and hyperthermia with metabolic acidosis during recovery and was euthanised 3 days after surgery. Horse 6 was excited, ataxic during recovery and developed myopathy and neuropathy. It stood up two hours after the end of the procedure with the assistance of the rope recovery system and a sling. This horse was subsequently euthanised two weeks following the initial surgery after several episodes of recumbency and inability to stand up without the help of the sling. Horse 7 became very excitable during recovery, developed metabolic acidosis and neurological signs. Despite intensive medical therapy for four hours following anaesthesia, no improvement was noted and the decision was taken to euthanise her. Horse 8 had a smooth recovery and stood up; however, 30 min later the horse suddenly became very excited, reared on its hind limbs and scrambled against the wall. It was sedated with romifidine and butorphanol but remained excited and developed possible malignant hyperthermia. Due to the poor prognosis of the initial complaint and complications the horse was euthanised. Horse 9 (ASA III) presented with abdominal pain and underwent exploratory laparotomy surgery. The horse developed severe myopathy and possible neuropathy in recovery. She was unable to stand and was euthanised the day after surgery. The following three horses were euthanised due to the severity of the musculoskeletal injury suffered. Horse 10 (ASA III) suffered an open luxation of the hock during recovery. Horse 11 (ASA IV) fractured her tibia in recovery and was euthanised. Horse 12 (ASA II) had a history of recurrent airway obstruction and was very excited during induction and recovery. She sustained a fracture of the right third metacarpal bone necessitating euthanasia.

## Minor complications

Eighteen horses suffered minor complications during the recovery (Table 3); none of them resulted in death. Five of the total cases (0.08%) were associated with failure of equipment and/or technique (loose head collar, tail hair breakage, tail knot slippage, facial paralysis).

## Discussion

To the authors knowledge this is the first retrospective study that documents specific problems occurring in the anaesthesia recovery period in horses. This large single-centre study reports a low minor complication and mortality rate when horses were sedated prior to recovery and assisted to stand with the head and tail rope system described above. This rope recovery system is suitable for use in any equine practice with a recovery stall.

A retrospective study published in 1993 focused on the identification of complications related to equine anaesthesia, with an emphasis on the recovery period [7]. In that study, the authors reported an overall complication rate of 1.4% (19/1314) and a mortality rate in recovery of 0.6% (8/1314) [7]. Eighteen horses developed problems whilst in recovery, mainly due to myopathy, neuropathy and/or fractures [7]. Dugdale’s recent study [8] documented intra-operative mortality during equine anaesthesia and, in addition, the complications which occurred in 4% of horses (58/1416) during the recovery period (from the time the horse entered the recovery box to the time it returned to its stall) were described. Complications were divided into mild (1.62%; 23/1416), moderate (0.56%; 8/1416), major (0.92%; 13/1416), and those horses that died or were euthanised due to anaesthetic recovery complications (0.99%; 14/1416). The authors identified factors that contributed to poor quality recovery or conversely improved recovery quality [8]. In neither of the above studies was an assisted recovery system used.

While we appreciate the difference in caseload, anaesthetic protocols and horse population between our study and that of Dugdale et al. [8], fewer horses experienced complications during recovery in our large study i.e. the overall complication rate was 0.51% (30/5852) with a 0.20% mortality/euthanasia rate (12/5852) when the one-man (head and tail) rope system was used.

We classified complications in our study as major (12 horses) or minor (18 horses). Of the major complications we reported, three suffered musculoskeletal injuries (0.05%) (2 fractures and 1 joint dislocation). This type of complication could be *truly* associated with a failure of the system to prevent lethal injuries, as the other complications i.e. myopathy and systemic disease, usually develop intra-operatively but only become evident during recovery period. Four of these horses were mares and considered geriatric [19] (range 15–19 years of age). It has previously been suggested that brood mares are at an increased risk of developing orthopaedic problems following anaesthesia because of decreased bone strength [20]. Records from the practice did not indicate if mares were pluriparturient or not and therefore we cannot deduce whether other factors unrelated to their age influenced the outcome. Notwithstanding this limitation, the occurrence of problems in older mares concurs with other reports [6, 20]. These animals may require greater assistance than this rope recovery system can provide and the authors suggest that mares of this age or older should be recovered with a more supportive system such as a sling (e.g. ‘Anderson Sling’ or ‘Large Animal Lift’). In addition, it may be beneficial if improved methods of assessing bone density in horses are developed so that risk may be quantified pre-operatively [20, 21]. In the meantime, owners should be informed of the increased risk of poor recovery and fractures in older mares.

Four horses experiencing major complications were described as excited or stressed during recovery and the rope recovery system may have adversely affected recovery quality in these animals. Previous studies identified temperament as a critical factor influencing recovery quality where horses with higher temperament scores (i.e. less placid) had worse recoveries [22, 23]. Unfortunately, in this practice, temperament was not scored, and non-docile individuals were not identified in advance. It is possible that the use of this rope recovery system may not be well suited to excitable or unhandled horses. Alternatively, identification of nervous horses and the judicious use of acepromazine and alpha_2_ agonists for premedication [2] and during recovery [9] may further reduce the risk of complications during recovery. Training on the ropes prior to induction of anaesthesia may also improve acceptance of this rope recovery system in nervous horses.

In our study five horses had major or minor complications following facial and /or dental surgery: this seems to be an over-representation given the caseload in this practice (60% orthopaedic, 34% soft tissue, 6% wound repair). It is unclear whether animals undergoing procedures of the head experience a greater degree of pain or disorientation in recovery, or if the rope recovery system (i.e. head collar with attached rope) resulted in an increased pressure on the head. Two of these horses were euthanised: one horse underwent a tooth extraction and the other had an invasive maxillary cyst removed. Both of these animals received analgesia with morphine and detomidine CRI; however, in neither of them were nerve blocks performed. Wilson et al. reported the use of detomidine CRI for standing chemical restraint and analgesia of horses undergoing surgical procedures [24]. Interestingly, the authors noted that head surgeries required additional doses of sedative and analgesic drugs [24]. They concluded that detomidine CRI alone did not provide adequate analgesia for procedures of this region of the body. Parviainen and Trim [25] also reported that horses undergoing ocular surgery (enucleation or surgery of adnexa/eye) had worse recoveries than horses undergoing general anaesthesia for splint bone excision. They proposed that the poor quality of recovery was related to pain from ocular surgery, or in some animals the sudden loss of vision in one eye causing disorientation. We suggest that inadequate analgesia may contribute to a poorer quality recovery in horses undergoing procedures of the head. Therefore, we recommend a multi-modal analgesic technique using opioids, appropriate nerve blocks with local anaesthetics (where possible), anti-inflammatory drugs and alpha agonists-adrenoceptor agonist infusions for analgesia, and sedation during the recovery period of this type of cases.

In five cases where minor complications occurred this was attributed to rope, equipment and/or technique malfunction (loose halter, facial nerve paralysis, tail hair breakage, tail support failure). Some of these minor complications could have been easily avoided. For example, it is essential that a variety of different sized head collars are available to ensure there is an appropriate size that fits the horse population; this avoids the loss of restraint/support when the horse attempts to stand if the head collar is loose. Facial nerve paralysis due to the head collar can be avoided by padding the area between the head collar and the facial nerve of the dependent side of the head; alternatively, an inflated tyre tube can be positioned underneath the horse’s head, and only head collars without metallic buckles should be used. This type of recovery system cannot be used effectively if the tail hair is of poor quality or the tail is very short. Tail hair breakage can be avoided by releasing the tension of the tail rope once the horse is standing, since some horses fight or resist the ropes when they stand up. Tail support failure can be avoided by ensuring an appropriate knot and use of rope material that does not slip. Horses should not be left unattended once they are standing until ropes are removed (usually less than 30 min after standing). It is recommended that the integrity of the system is assessed daily (ropes, belay, pulleys, and halter) and maintenance carried out on a regular basis. Finally and very importantly, personnel using this equipment should have appropriate training prior to using it.

A number of other factors may contribute to the low complication rate we report here. Firstly, at this practice, most horses received an alpha_2_ agonist CRIs during anaesthesia: this has not been reported in earlier anaesthetic mortality studies. These drugs are known to have a significant anaesthetic-sparing effect [26–28], which may contribute to a reduction in the risk of complications during recovery. In addition, all horses received sedation in recovery, and the use of sedation early in recovery is associated with significantly better recovery scores [8]. We speculate that the use of the described rope recovery system in combination with sedative drugs ensures better recovery quality, although a prospective study with a control group would be required to confirm this. Moreover, the urinary bladder of all our horses was emptied prior to recovery; this may lessen discomfort in the recovery period, prevent precocious attempts to stand and improve the horse’s footing as the floor remains free from urine. In addition, the small recovery box used at this practice may be of benefit although this is not supported by the current literature.

The majority of horses experiencing complications in our report were Warmbloods and this in agreement with the predominant type of horse presented to this clinic. Woodhouse et al. [10] reported that Arabian horses had a poorer quality recovery when compared with other types of horse, arguably due to a more unpredictable temperament. It remains unclear whether Warmblood horses are calmer and more stoical than other horse breeds. Further studies using the system with a greater diversity of horse types and temperaments may help to answer this question.

Currently assisted recovery is often employed to improve outcome, predominantly in higher risk cases (e.g. fracture repair) [2]. However, no study has truly determined the effectiveness of assisted recovery in reducing complications. The use of assisted recovery techniques varies geographically. In the largest multi-centre equine anaesthetic mortality study no differentiation between the use of assisted and unassisted recovery and the associated incidence of mortality was reported [2]. In that study, approximately 32% of complications were of musculoskeletal origin [2]. In the largest single-centre equine mortality study Bidwell et al. [6] reported an overall intra-operative mortality of 0.12%, with this rising to 0.24% when problems post-operatively (up to 7 days after anaesthesia) were included. In the latter study a form of assisted recovery was used routinely, with ropes attached to the head collar and the tail and recovery assisted by two people positioned inside the recovery box [6]. The latest anaesthetic mortality study by Dugdale et al. [8] documented a 1% mortality rate in recovery where horses were allowed to recover unassisted (mostly following orthopaedic procedures). Notwithstanding the differences between our studies and that of Dugdale et al., we suggest that the routine use of a rope-assisted recovery system such as that described herein may be associated with fewer orthopaedic complications.

## Conclusion

In conclusion, we report a complication rate of 0.51% and a mortality rate of 0.20% in horses recovering from general anaesthesia when a purpose designed one-man (head and tail) rope recovery system was used in 5852 horses in a private equine clinic. The low mortality rate (0.15%) recorded in Group 2 where the rope recovery system failed to prevent a fatal complication could, at least in part, be attributed to routine sedation just prior to recovery and to the type of rope recovery itself. Older mares are at higher risk of suffering fractures in recovery and the use of this rope recovery system may not be sufficient to eliminate these complications in that sub-population. Based on these data the authors propose that the use of this rope recovery system contributes to an improvement in recovery quality when compared to studies where recovery was unassisted and may compare very favourably with other assisted recovery systems. However, the retrospective nature of this study, the variety of drugs used, the lack of control group and randomisation are limitations of the study. There is an inherent bias in the breed of horses since Warmbloods were over-represented in this clinic. A prospective multi-centre study with a greater variety of horse breeds and procedures would allow a more objective assessment of the one-man (head and tail) rope recovery system.
